# Supplementary material for: Context-Dependent Distinct Roles of SOX9 in Combined Hepatocellular Carcinoma–Cholangiocarcinoma
Source: Cells. 2024 Aug 29;13(17):1451. doi: 10.3390/cells13171451 (PMC11394107; doi:10.3390/cells13171451)
Supplement: Supplementary file 1 [file cells-13-01451-s001.zip › cells-3094217-File S1.pdf]

ONLINE SUPPLEMENT

Table of content:

1. **SUPPLEMENTARY METHODS**
2. **SUPPLEMENTARY TABLES (Table S1-S3)**

1. SUPPLEMENTARY METHODS

Animal Models of Intrahepatic Cholangiocarcinoma. The constructs used for mouse SB-HDTVI, including *pT3-EF1α, pT3-EF1α- myrAkt-HA* (mouse), *pT3-EF1α-YAP1 S127A* (human), *pT3-EF1α-NRAS V12 caggs, pCMV-empty*, *pCMV-Cre,* and *pCMV-sleeping beauty transposase* (SB) were generated or have been described elsewhere [10,49,50]. All the plasmids used for *in vivo* experiments were purified using the Endotoxin Free Maxi Prep kit (Sigma-Aldrich). Six-to-eight weeks old mice were randomized into groups and subjected to the sleeping beauty transposon-transposase and hydrodynamic tail vein (SB-HDTVI) protocol as described previously [49]. Briefly, 10 μg *pT3-EF1α-myrAkt-HA*, *pT3-EF1α-YAP1 S127A* or 10 μg *pT3-EF1α-myrAkt-HA* and 20 μg of *pT3-EF1α-NRAS V12 caggs,* and 40 μg of *pCMV-Cre (or pCMV-empty), and/or newly generated CMV-LSL-Cas9;U6-sg-Sox9* plasmid (described below) along with the transposase in a ratio of 25:1 were diluted in 2 ml of normal saline (0.9% NaCl), filtered through 0.22μm filter (Millipore), and hydrodynamically injected into the lateral tail vein of mice. All animals were sacrificed between 2-6 weeks of plasmids injections unless otherwise indicated.

Patient data. All human tissue samples were provided by Pittsburgh Liver Research Center's (PLRC's) Clinical Biospecimen Repository and Processing Core supported by P30DK120531 under approved Institutional Review Board STUDY19070068. TMAs were constructed from archival formalin-fixed paraffin-embedded tissue blocks from 108 cholangiocarcinoma patients seen at the University of Pittsburgh Medical Center and were also obtained from PLRC's CBPRC supported by P30DK120531. All tumor hematoxylin and eosin (H&E) stained slides were reviewed, and representative areas were carefully selected for tissue microarray construction. Two, random 1.0 mm-sized cores were punched from each patient's tumor and harvested into recipient blocks. The demographics and additional information of these cases are included in Supplementary File S2. The TMA were stained manually using antibody against SOX9 (EMD Millipore) and YAP1 (Cell Signaling) as described in IHC sections. Whole slide image capture of the tissue microarray was acquired using the Aperio XT slide scanner (Aperio Technologies). The staining was evaluated and scored by anatomic pathologist (A.S.). Staining for SOX9 and YAP was scored either as 0 (negative), 1+ (mostly cytoplasmic staining or very weak staining in CC tumor cells), or 2+ (strong positive nuclear staining in CC tumor cells). For all 2 markers, the scores for different tissue sections from each patient were averaged to get a single score per patient (Supplementary File S2). Mean scores greater than or equal to 1.5 were considered "HIGH" and mean scores less than 1.5 were considered "LOW/NEGATIVE". All scores for each individual section are included in Supplementary File S2.

Constructs *CRISPR/Cas9-sg-Sox9* transposon vectors and overexpression vectors

We subcloned CMV-LSL-Cas9-P2A-EGFP fragment from the LSL-Cas9-Rosa26TV plasmid (Addgene #61408) by digesting with PacI (NEB# R0547L) and NsiI (NEB# R0127S), followed by purification using a QIAquick PCR & Gel Cleanup Kit (Qiagen# 28506). Next, we amplified a cassette from the CRISPR-SB plasmid (Addgene #177936) with the High Fidelity PCR EcoDry™ Premix (TaKaRa#639280). This cassette included plasmid replication elements, an SB-transposon, and a sgRNA scaffold-linker-U6 promoter. We assembled this cassette with the CMV-LSL-Cas9-P2A-EGFP fragment using the NEBuilder HiFi DNA Assembly Master Mix (NEB#E2621L).

Cloning CRISPR sgRNA for the target genes
We determined the *CRISPR sg-Sox9* target sequences using the CHOPCHOP online tool (https://chopchop.cbu.uib.no). Following this, we ordered two complementary single-strand oligonucleotides of the target sequence from IDT with specific overhangs: one with a 5'-CACC overhang and the other with a 3'-ACCC overhang. To insert these sgRNA target sequences into construct, we first phosphorylated them using T4 PNK (NEB#M0201S), then denatured and annealed them to form double-stranded oligos. Next, we linearized the CRISPR/Cas9-sgRNA transposon plasmid with BbsI (NEB# R0539L) and inserted the double-stranded oligo using the NEB Quick Ligation™ Kit (NEB#M2200S). To improve our chances of achieving a knock-out, we incorporated an additional CRISPR sgRNA target sequence with sgRNA scaffold-linker-U6 promoter cassette into the final construct. This was done by inserting another double-stranded oligo into the CRISRP-SB plasmid, which had been digested with BbsI (NEB# R0539L). We then amplified this additional sgRNA sequence-linker-U6 promoter cassette and integrated it into the previous *CRISPR/Cas9-sgRNA* transposon plasmid containing the previous CRISPR sgRNA target sequence. This integration utilized the NEBuilder HiFi DNA Assembly Master Mix (NEB#E2621L), with the linearization performed by PacI (NEB# R0547L). For details on all constructs and primers used in this project can be obtained upon request.

Immunohistochemistry (IHC). Mouse liver tissues were fixed for 48 h in 10% neutralized formalin (Fisher Chemicals), transferred into 70% ethanol and then dehydrated and embedded in paraffin. For IHC, formalin-fixed sections were deparaffinized in graded xylene and ethanol and rinsed in PBS. For antigen retrieval, samples were microwaved for 12 min in pH 6.0 sodium citrate buffer (HA-tag, panCK, SOX9) or pH 9.0 Tris-EDTA buffer (p-AKT, CK19), or were pressure cooked for 20 min in pH 9.0 Tris-EDTA buffer (YAP1 and HNF4α). After cooling, samples were placed in 3% H_2O_2 (Fisher Chemicals) for 10 min to quench endogenous peroxide activity. After washing with PBS, slides were blocked with Super Block (ScyTek Laboratories) for 10 min. Sections were incubated for overnight at 4C with the primary antibodies (listed in table below). Sections were then incubated with species-specific biotinylated secondary antibodies (EMD Millipore, listed in table below) for 1 h, at room temperature. Sections were incubated with Vectastain ABC Elite kit (Vector Laboratories) and signal was detected with DAB Peroxidase Substrate Kit (Vector Laboratories) followed by quenching in distilled water for 5 min. Slides were counterstained with hematoxylin (ThermoFisher Scientific), dehydrated to xylene (Fisher Chemicals) and coverslips applied with Cytoseal XYL (ThermoFisher Scientific). To assess cell death, TUNEL staining was performed using the ApopTag Peroxidase In situ Detection kit (Milipore, cat# S7100) according to the manufacturer's instruction.

Immunofluorescence. Paraffin embedded liver sections (5 μm thick) were deparaffinized using xylene (Fisher Chemicals) and rehydrated by incubating the slices in ethanol (100% and 95% v/v, each 3x5 min) and washed in PBS. Heat-induced epitope retrieval was performed for 20 min using a pressure cooker with pH 6.0 sodium citrate buffer. Sections were washed in PBS, permeabilized for 5 minutes with PBS/0.3% Triton X and blocked with PBS/0.3% Triton X/10% bovine serum albumin (BSA) for 45 minutes at room temperature. Sections were incubated with primary antibodies in PBS/0.3% Triton X/10% BSA overnight at 4C. At the end of the incubation, sections were washed thrice and incubated with fluorochrome-conjugated secondary antibodies in

PBS/0.3% Triton X/10% BSA for 1h at room temperature, then washed in PBS/0.1% Triton X 3 times. Liver sections were mounted using Prolong Gold Antifade w/DAPI (Invitrogen) and pictures were acquired using LSM700 confocal microscope the and Zen Software (Zeiss).

RT-qPCR analysis.
Whole liver was homogenized in TRIzol™ (Thermo Scientific, Cat# 15596026), treated with chloroform, and nucleic acid was precipitated with isopropanol. Cellular DNA was digested with DNA-free™ Kit (ambion, AM1906), and RNA was reverse-transcribed into cDNA using SuperScript® III (Invitrogen, 18080-044). Real-time PCR was performed in technical triplicate on a StepOnePlus™ Real-Time PCR System (Applied Biosystems, Cat# 4376600) using the Power SYBR® Green PCR Master Mix (Applied Biosystems, 4367660). Target gene expression was normalized to the average of two housekeeping genes (Gapdh and Rn18s), and fold change was calculated utilizing the ΔΔ-Ct method. Primers are listed in Table below.

RNA-Seq Analysis. For each group (*Akt-YAP1 Sox9* WT and *Akt-YAP1 Sox9* LKO), two mouse liver samples were processed for RNA-seq analysis. RNA samples from livers were used to generate library using TruSeq kit from Illumina [51,52]. We used in-house HiSeq2500 platform and sequenced 200 million reads to accurately quantify genes & transcripts [53]. Raw sequencing data was analyzed by FastQC for quality control [54]. Low quality reads or adapter sequences were trimmed out by Trimmomatic [55]. After pre-processing, sequenced reads were aligned to mouse reference genome mm10 by HISAT2 aligner [56]. Read counts for each gene were then quantified by HTSeq [57]. All the pipelines were run by default parameter settings. RNA-seq data have been submitted to the online database gene expression omnibus (GEO) accession ID: GSE200472. Differential expression (DE) analysis was performed to compare *Akt-YAP1 Sox9* WT versus *Akt-YAP1 Sox9* LKO. Based on the read counts, DE tests were performed by R package 'DEseq2' [58] and top DE genes were selected by absolute fold-change greater than 1 and FDR=0.05. These DE genes were then used as input for Ingenuity Pathway Analysis (IPA)® to call pathways that were significantly enriched with FDR=0.1.

Bioinformatic comparison between mouse and human models. To further investigate how the mouse models may mimic a subset of human liver cancer including CCA or HCC, public human transcript data were collected to compare with the gene signature obtained from the mouse models. For HCC, LIHC TCGA database was assessed similarly. After pre-processing, gene expression data were analyzed by R package 'limma' test [59] and top differentially expressed genes were selected by absolute fold-change greater than 1 and FDR=0.05 (same criteria as the mouse model). Top DE genes from human study were further used to detect significantly enriched pathways by IPA® software with FDR=0.1. To test the molecular similarity between our mouse *Akt-YAP1* liver cancer model and the human studies, three publicly available human CCA the cancer genome atlas program (TCGA) dataset was analyzed by three comparisons. (1) Pathway enrichment analysis. Top enriched pathways detected by mouse model and human studies were detected independently and compared. (2) Gene signature analysis. Top DE genes from mouse models were converted to human homologous genes by Mouse Genome Database (MGD) [60]. These genes were then applied into the human studies to check their expression signatures. (3) Signature prediction. Positive prediction of gene signatures tested in

this study has been calculated by Nearest Template Prediction (Gene Pattern module), [61] as previously described [21]. For CCA studies, each patient was also assigned to either the Proliferation or Inflammation class based on the results of unsupervised clustering as previously described [62]. Additional signatures assessed were for Notch activation [63] and hepatic stem-cell like group of ICC patients [64]. Any significant correlation to a subclass was noted by p value ($p<0.05$).

Statistical Analysis. For all mouse experiments, sample size was pre-determined based on previous literature describing SB-HDTVI-mediated liver carcinogenesis [49]. Accordingly, littermates were randomized into groups for HDTVI and managed throughout the course of treatment in a non-blinded manner. All subsequent molecular, immunohistochemical, and immunofluorescence analysis was performed in a blinded manner. All confidence intervals shown on the bar plots are presented as mean ± standard error of mean (SEM). Differences in mean values of liver volume and LW/BW ratio were analyzed by one-way ANOVA assuming normal Gaussian distribution with Geisser Greenhouse posttest correction. $p<0.05$ was considered significant (*), $p<0.01$ was considered highly significant (**), $p<0.005$ was considered extremely significant (***), and so on. All statistical analysis on patient samples has been included in the results section and respective p-values were included in the pertinent text and figure legends. All statistics were performed using GraphPad Prism 10.0 (GraphPad Software) or R software.

2. Supplementary Tables

Table S1: List of antibodies for IHC and IF in this study.
Primary Antibodies:

Target	Species	Dilution	Source	Catalog Number
HA-tag	Mouse	1:50 (IHC)	Cell Signaling	CS2367
Pan-CYTOKERATIN (panCK)	Rabbit	1:200 (IHC)	Dako	Z0622
SOX9	Rabbit	1:200 (IF)<br>1:2000 (IHC)	EMD Millipore	Ab5535
YAP1	Rabbit	1:100 (IF and IHC)	Cell Signaling	CS14074
HNF4α	Rabbit	1:100 (IHC)	Cell Signaling	CS3113
Ki-67	Goat	1:100 (IF)	Santa Cruz	sc-7846
CK19	Rat	1:10 (IF)	DSHB	TROMA III

Table S2: Secondary Antibodies:

Secondary Antibody	Species	Source	Catalog Number
Donkey anti-Rabbit IgG Biotin	Donkey	EMD Millipore	AP182B
Goat anti-Mouse IgG (H+L) Biotin	Goat	EMD Millipore	AP181B
Alexa-Fluor 555 Donkey anti-Rabbit IgG (H+L)	Donkey	Invitrogen	A31572
Alexa-Fluor 488 Donkey anti-Rat IgG (H+L)	Donkey	Invitrogen	A21208
Alexa-Fluor 647 Goat anti-Mouse IgG (H+L)	Goat	Invitrogen	A21236
Alexa-Fluor 488 Donkey anti-Rat IgG (H+L)	Donkey	Jackson Immuno Research Labs	712-545-153 (NC0236082)

Alexa-Fluor 647 Goat anti-Mouse IgG (H+L)	Donkey	Jackson Immuno Research Labs	715-605-151 (NC0299851)

Table S3: Nucleotide sequence of primers used in this:

Gene	Primer	Nucleotide sequence (5' to 3')
Sox9	forward	CAGGCAAGAATTGGGCAAAG
Sox9	reverse	CCTCCCAACACGCAGTAAA
CK19	forward	GACCTGGAGATGCAGATTGAG
CK19	reverse	GCTCCTCAGGGCAGTAATTT
Hnf1β	forward	AACCAGCCGGGAAACAATGA
Hnf1β	reverse	CTCCCGACACTGTGATCTGC
EpCAM	forward	AACACAAGACGACGTGGACA
EpCAM	reverse	GCTCTCCGTTCACTCTCAGG
Hnf4α	forward	ATGGATATGGCCGACTACAGCGCT
Hnf4α	reverse	ACAGCTCGAGGCTCCGTAGTGTTT
Afp	forward	TCGTATTCCAACAGGAGG
Afp	reverse	AGGCTTTTGCTTCACCAG
Tat	forward	GCTATGCCCCATCTATCGGC
Tat	reverse	GCAGCCACTCGTCAGAATGA
Tdo2	forward	CTGGGGGATCCTCAGGCTAT
Tdo2	reverse	TGTCACTGTACTCGGCTGTG
Gapdh	forward	AACTTTGGCATTGTGGAAGG
Gapdh	reverse	ACACATTGGGGGTAGGAACA
Rn18s	forward	GTAACCCGTTGAACCCCATT
Rn18s	reverse	CCATCCAATCGGTAGTAGCG
